# Epidemiology of Neonatal Sepsis in Haiti

**DOI:** 10.1017/ash.2024.295

**Published:** 2024-09-16

**Authors:** Mentor Lucien, Theline Cantave, Hudline Vilfrard, Pascale Pierre, Carla Nelson

**Affiliations:** Quisqueya University; Université Notre-Dame d'Haïti

## Abstract

**Introduction:** Neonatal sepsis (NS) is a global public health concern, particularly affecting developing countries. Challenges in diagnostics, more specifically, culture and antimicrobial susceptibility testing hinder effective management of the disease. **Objective:** This study aims to evaluate the burden, describe the management, and assess the evolution of NS in a hospitalized pediatric population in Haiti. **Methods:** A retrospective cohort study from January 2013 to December 2018 at La Paix University Hospital was conducted. All-cause hospitalizations and deaths were extracted from hospital’s Neonatology Unit records and were used to derive data regarding hospitalization and death among patients under 28 days with NS. Clinical and laboratory data were extracted from the patients’ medical records. **Results:** Out of 2,424 post-childbirth hospitalizations, 1,590 involved sepsis. The percentage of hospitalization due to NS was approximately 69% and the percentage of deaths, 65%. The mean age of patients with NS was 3.75 days (0 - 22 days), with a slight male predominance (55%, p < 0.001). Peaks were observed from May to August (p = 0.02). Early NS cases (NS in patients aged less than 7 days) were most prevalent (86%, p < 0.001). Specimen culture and antimicrobial susceptibility testing was less frequent (7%) than complete blood count usage (65%). Findings regarding blood count included leukopenia (3%), thrombocytopenia (30%). A positive CRP and acute renal failure were noted in 76% and 21.7% of cases, respectively. The average hospital stay was 7.3 days. With regards to treatment, 73% of patients received a 2-drug antimicrobial therapy (ampicillin-gentamycin) and 22% received a 3-drug antimicrobial therapy (ampicillin-gentamycin-cefotaxime). Of all newborns hospitalized for NS, 49% received empirical antibiotic therapy within 3 hours of admission. **Conclusions:** This research highlights NS as a public health emergency in Haiti. The study advocates for improved access to culture and antibiotic susceptibility testing and emphasizes the impact of timely antibiotic administration. The findings of this study serve as a baseline for informing policymakers and medical practitioners dedicated to improving existing conditions of neonates in Haiti. Suggested targeted interventions include preventive measures during prenatal visits, strengthening laboratory capacities, improving infection prevention and control measures, and developing antimicrobial stewardship programs.

